# Inter-Model Warming Projection Spread: Inherited Traits from Control Climate Diversity

**DOI:** 10.1038/s41598-017-04623-7

**Published:** 2017-06-27

**Authors:** Xiaoming Hu, Patrick C. Taylor, Ming Cai, Song Yang, Yi Deng, Sergio Sejas

**Affiliations:** 1 Department of Atmospheric Sciences, Sun Yat-sen University, Guangzhou, China; 2 Department of Earth, Ocean & Atmospheric Sciences, Florida State University, Tallahassee, Florida USA; 3 NASA Langley Research Center, Climate Science Branch, Hampton, Virginia USA; 4 School of Earth and Atmospheric Sciences, Georgia Institute of Technology, Atlanta, Georgia USA

## Abstract

Since Chaney’s report, the range of global warming projections in response to a doubling of CO_2_—from 1.5 °C to 4.5 °C or greater —remains largely unscathed by the onslaught of new scientific insights. Conventional thinking regards inter-model differences in climate feedbacks as the sole cause of the warming projection spread (WPS). Our findings shed new light on this issue indicating that climate feedbacks inherit diversity from the model control climate, besides the models’ intrinsic climate feedback diversity that is independent of the control climate state. Regulated by the control climate ice coverage, models with greater (lesser) ice coverage generally possess a colder (warmer) and drier (moister) climate, exhibit a stronger (weaker) ice-albedo feedback, and experience greater (weaker) warming. The water vapor feedback also inherits diversity from the control climate but in an opposite way: a colder (warmer) climate generally possesses a weaker (stronger) water vapor feedback, yielding a weaker (stronger) warming. These inherited traits influence the warming response in opposing manners, resulting in a weaker correlation between the WPS and control climate diversity. Our study indicates that a better understanding of the diversity amongst climate model mean states may help to narrow down the range of global warming projections.

## Introduction

Since Charney’s report^1^, the range of projection of global warming in response to double of CO_2_ has largely remained unchanged: from 1.5 °C to 4.5 °C or greater^2–7^. An essential question is thus, why different climate models, under the same anthropogenic forcing, produce different amounts of global mean surface warming. A definitive answer to this question is central to the current scientific and societal deliberation, and will alter ongoing adaptation and mitigation efforts and future climate policy^8, 9^. Efforts to address this question often focus on the climate model response and feedbacks^10–16^, as a clear mathematical framework based on energy balance describes the relationship between climate feedbacks and surface warming. This ‘climate feedback lens’ has zoomed in on cloud feedback and revealed specifically marine stratocumulus low clouds as the largest contributor to climate change uncertainty^17–19^. This conventional view holds radiative feedbacks as the sole culprit for the global warming projection spread (WPS) among different climate models’ equilibrium (or transient) response to the same anthropogenic greenhouse radiative forcing, while directing little attention to the diversity among model control climates. Several studies have revealed that the control climate sea ice characteristics regulate the ice-albedo feedback^20–26^, as more extensive sea ice coverage contributes to a stronger ice-albedo feedback due to an increased potential for ice melt^20, 23^. Therefore, control climate influences a model’s response to a radiative forcing by modulating the ice-albedo feedback strength.

Here we argue that it would be more fruitful to distinguish the climate feedback diversity that is strongly dependent of models’ control climate state from the intrinsic climate feedback diversity that is independent from the control climate state. Both types of climate feedback diversities are rooted on the diversity in physical and dynamical parameterizations^27, 28^. Even different parameterizations of various sub-grid processes could compensate one another to reach the same control climate state, they might not be able to do so when subject to an external climate forcing, giving rise to the second type of climate feedback diversity. Furthermore besides the lack of compensating effects between different parameterizations, causing control climate diversity as well as the associated climate feedback diversity, control climate state diversity can also be due to the existence of multiple equilibrium states for the same energy input to the climate system^29, 30^. Such diversity in control climates, under the same external forcing, may explain a portion of the uncertainty in global warming projections. In this study, we focus on the evidence for the climate feedback diversity that is inherited from the control climate diversity. We wish to further demonstrate that besides the ice coverage diversity, differences in models’ other variables describing the control climate state, such as water vapor content, can also contribute to the climate feedback diversity. The compensating effect of climate diversity associated with different climate variables inherited from control climate diversity makes the relationship between WSP and control climate diversity less obvious or obscured. The recognition of the inheritance of the WPS from the diversity of model control climate states provides a new pathway for understanding and reducing model uncertainty.

## Definition of key climate variables

We consider 31 140-year CMIP5 (the phase 5 of the Coupled Model Intercomparison Project) climate simulations under the same solar energy input plus a steady, 1% per year CO_2_ increase starting from the pre-industrial CO_2_ concentration level of 280 PPMV (the 1pctCO2 experiments, Supplementary Table S1). We consider eight key climate variables (Supplementary Tables S2 and S3): (i) surface temperature (T), (ii) vertically integrated atmospheric water vapor content (q), (iii) vertically integrated cloud water/ice content (CL), (iv) area covered by ice/snow (IC), (v) the difference between the net downward radiative fluxes at TOA and the net energy flux at the surface whose spatial pattern measures the strength of the total energy transport by atmospheric motions (DYN), (vi) evaporation (E), (vii) the difference between surface evaporation (E) and precipitation (E − P) whose spatial pattern measures the strength of atmospheric latent heat transport, and (viii) surface sensible heat flux (SH). Considered at the time of CO_2_ quadrupling (4 × CO_2_), the transient climate response (denoted as Δ) is defined as the difference between the perturbed and control climate states specified as the average over the last 10-year period minus the first 10-year period. For the sake of brevity, we use “{X_j_}” to denote a series of 31 values of X_j_, or {X_j_, j = 1, 2 …, 31}, where X_j_ is the departure in the jth experiment from the ensemble mean of the 31 1pctCO2 experiments of the climate mean or its change of a climate variable X (see Data and Methods for details). For an easy reference, we also refer to {X_j_} as the spread of X among the 31 experiments.

We use {<Δ*T*_*j*_>} (“< >” denotes the global mean) obtained from different models’ 1pctCO2 experiments as the individual models’ transient climate responses to CO_2_ quadrupling forcing and their numerical differences correspond to the warming projection spread (WPS). Besides the 31 values of <Δ*T*_*j*_>, we also consider changes in other 7 climate variables derived from these 31 1pctCO2 experiments. Specifically, {<Δ*q*_*j*_>} corresponds to the spread of the transient response in the global mean total atmospheric water vapor content, measuring the global water vapor feedback strength spread. Similarly, we use {<Δ*CL*_*j*_>}, {<Δ*IC*_*j*_>}, {<Δ|*DYN*_*j*_|>} (“| |” denotes the absolute value), {<Δ*E*_*j*_>}, {<Δ|*E*_*j*_ − *P*_*j*_|>}, and {<Δ*SH*_*j*_>}, respectively, to measure the spreads in the global cloud feedback, the global ice albedo feedback, the atmospheric energy transport feedback, the evaporation feedback, the hydrological cycle response, and in the surface sensible heat flux feedback. In short, the spreads of {<Δ*q*_*j*_>}, {<Δ*CL*_*j*_>}, and {<Δ*IC*_*j*_>}, represent the spread in the key thermodynamic feedback agents considered in the conventional partial radiative perturbation feedback analysis^12^, while the remaining 4 spreads collectively give rise to the lapse-rate feedback spread due to non-radiative feedback agents^31, 32^. See Data and Methods for correlation, partial correlation, and covariance analyses that relate the 31 values of <Δ*T*_*j*_> or the WPS, to the spreads in these climate feedback agents and to their mean values in the control climate state).

## Spreads in global warming projections, climate feedbacks, and control climate states

**Figure 1:**
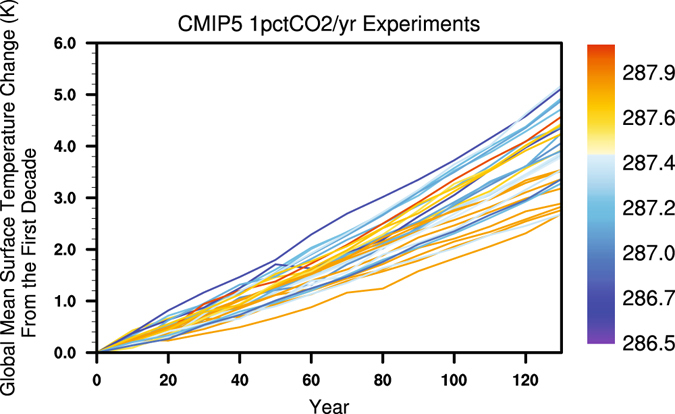
Time series of global mean surface temperature change of the 31 CMIP5 1pctCO2 experiments relative to their corresponding first 10-year averages (labeled as “Year 0” which has been set to zero for each curve). The color scheme for these 31 curves represents the global and time mean surface temperature of the first 10-year simulations of the 31 CMIP5 1pctCO2 experiments. The color scheme is arranged in such a way that the control climate state ranges from the coldest to the warmest as the color changes from blue to red.

**Figure 2:**
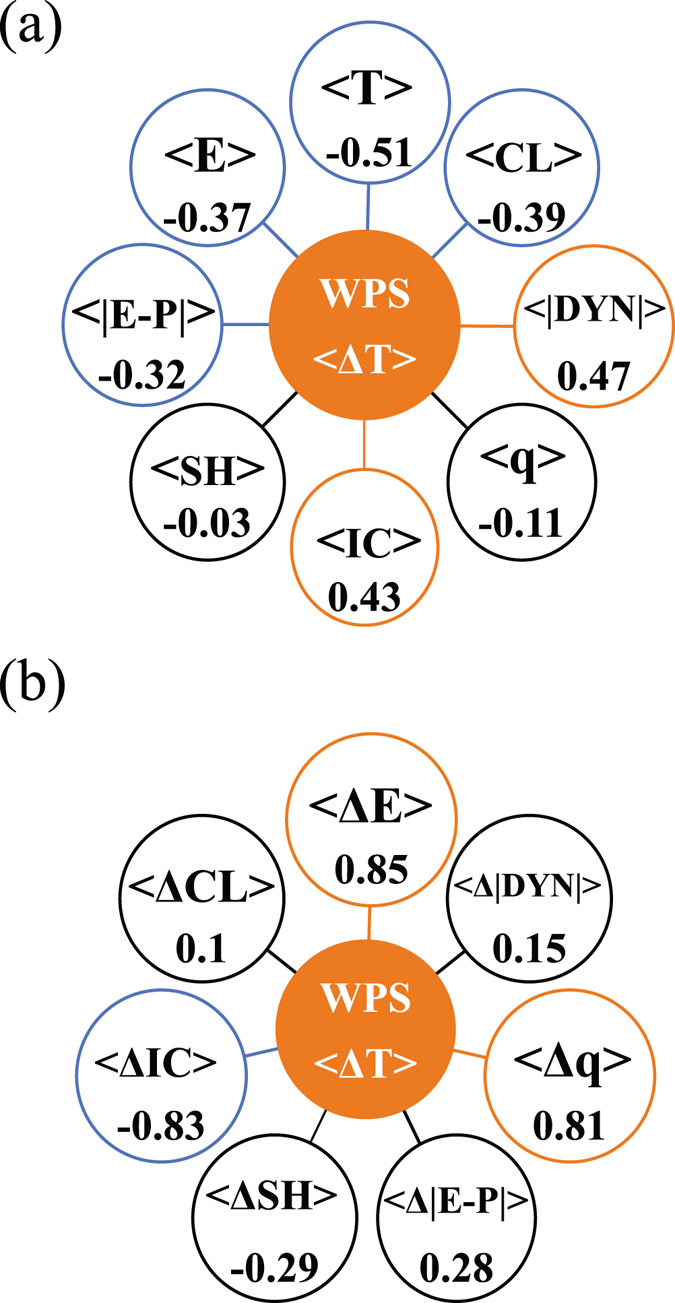
Correlation coefficients between the warming projection spread (WPS) and (**a**) spreads in the eight key control climate state variables, (**b**) spreads in the key climate variable transient responses to 4xCO_2_. Numbers in orange and blue colored (black) circles indicate the correlation coefficients (do not) exceed 90% confidence level.

Figure 1 shows {<Δ*T*_*j*_>} obtained from the 31 1pctCO2 experiments as a function of model integration time. The WPS among these 31 simulations emerges shortly after the simulation begins displaying a range of 2.5 °C to 5.2 °C at the time of 4 × CO_2_. Indicated by Fig. 2a, a significant portion of this WPS is explained by the diversity in key control climate variables. The largest correlation is found to be between {<*T*_*j*_>} and {<Δ*T*_*j*_>} (−0.51), implying colder models experience greater warming. Often accompanying colder <*T*_*j*_>, models with larger <*IC*_*j*_> have greater melt potential (Fig. 2a and Supplementary Fig. S2), which favors an enhanced ice-albedo feedback and thereby a stronger warming^12, 23^. The spread in dynamic energy transport also positively correlates (0.47; Fig. 2a) with WPS indicating that models with stronger poleward energy transport experience greater warming. Though weaker in magnitude, {<*E*_*j*_>}, {<|*E*_*j*_ − *P*_*j*_|>}, and {<*CL*_*j*_>} also show statistically significant correlations with {<Δ*T*_*j*_>}.

Indeed, spreads of individual climate feedbacks describe a significant portion of the WPS. The correlation between WPS and {<Δ*IC*_*j*_>} (−0.83; Fig. 2b) indicates that more ice melt relates to larger warming. Figure 2b also shows large correlations of {<Δ*E*_*j*_>} (={<Δ*P*_*j*_>}) (0.85) and {<Δ*q*_*j*_>} (0.81) with WPS; models with larger increases in {<Δ*E*_*j*_>}, {<Δ*P*_*j*_>} and {<Δ*q*_*j*_>} experience greater warming. Unlike Fig. 2a,b indicates no other statistically significant correlations besides those aforementioned.

## Two types of inherited traits from control climate states

**Figure 3:**
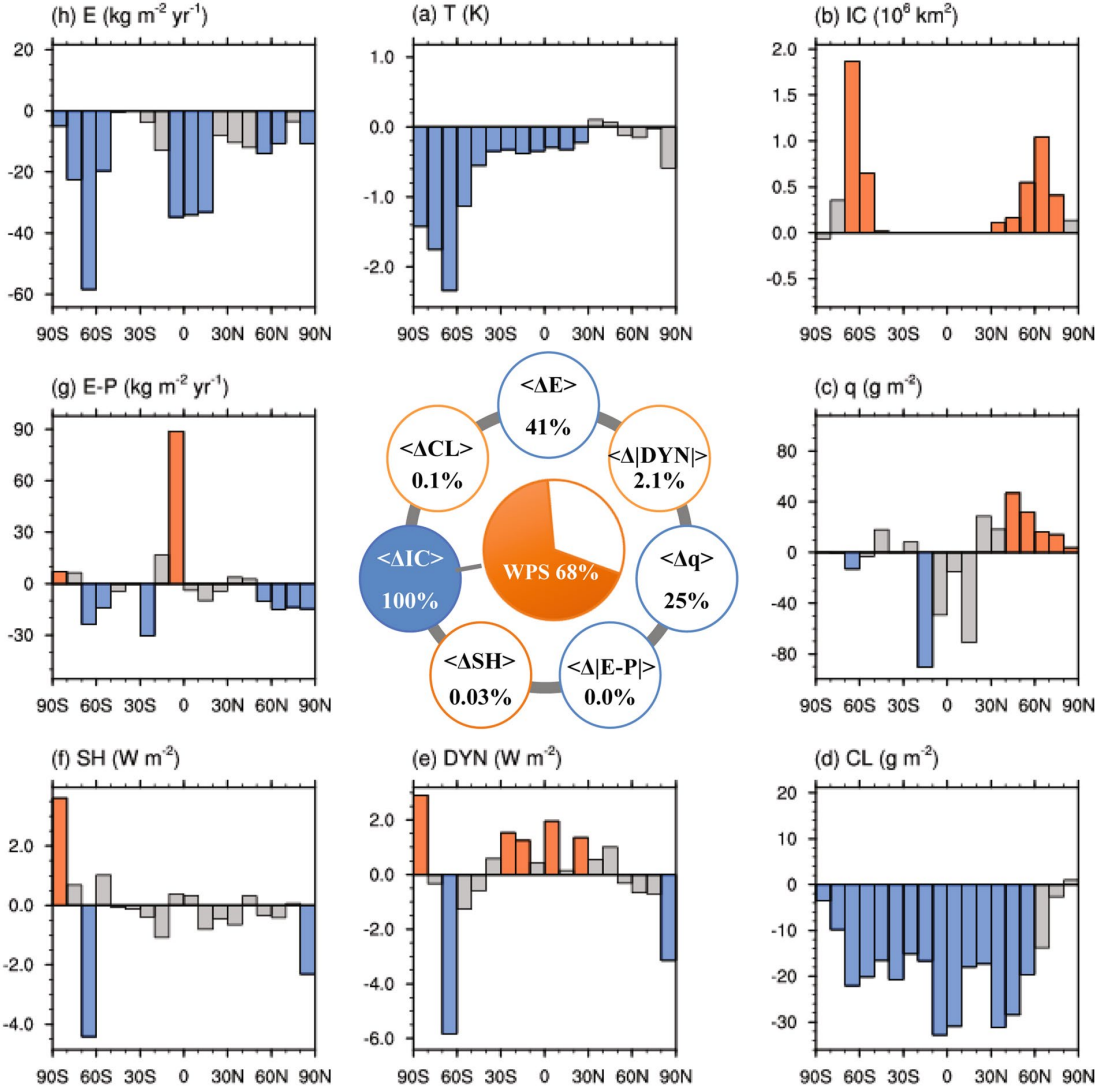
Latitudinal profiles (outer panels) of the regressed spreads of the zonal mean control climate states (**a**–**h**) against the projected spread in the change of total area coverage by ice/snow. (**a**) Surface temperature (*T* in units of K), (**b**) total area covered by ice/snow (*IC* in units of km^2^), (**c**) vertically integrated atmospheric water vapor content (*q* in units of g m^−2^), (**d**) vertically integrated cloud water/ice content (*CL* in units of g m^−2^), (**e**) net downward radiative fluxes at TOA which measures the strength of the total atmosphere-ocean energy transport (*DYN* in units of W m^−2^), (**f**) surface sensible heat flux (*SH* in units of W m^−2^), (**g**) difference between surface evaporation rate and precipitation rate (*E* − *P* in units of kg m^−2^ yr^−1^), and (**h**) precipitation rate (*P* in units of kg m^−2^ yr^−1^). The numbers inside the circles of the inner panel correspond to the percentage of the spread, in the global mean changes of the eight key climate state variables that can be explained by the spread in the change of total ice/snow area coverage. Orange and blue colored (grey) bars indicate the correlation coefficients (do not) exceed 90% confidence level.

The correlations in Fig. 2a suggest that the WPS is associated with the control climate diversity. Employing a series of partial regression analyses (see Data and Methods), we link the WPS to differences in climate feedbacks and then analyze the associations of feedback differences with control climate features. As indicated in Fig. 2b, {<Δ*IC*_*j*_>}, {<Δ*E*_*j*_>} (={<Δ*P*_*j*_>}), and {<Δ*q*_*j*_>} each exhibits a nearly identical high correlation with the WPS. It is seen that the association of the control climate spread with {<Δ*IC*_*j*_>} (Fig. 3) is most similar to that associated with the WPS (Fig. S2), compared to the other two possible permutations (Supplementary Fig. S3 for {<Δ*E*_*j*_>} and Supplementary Fig. S4 for {<Δ*q*_*j*_>}). This implies that the linkage of the WPS to the control climate spread can be explained more through the linkage of {<Δ*IC*_*j*_>} to the control climate spread than {<Δ*E*_*j*_>} and {<Δ*q*_*j*_>}, although their correlations with the WPS are about the same. Therefore, we choose <Δ*IC*_*j*_> as the starting point of the successive partial correlation analysis. Figure 3 (inner panel) demonstrates the interdependence of the climate response variables, indicating that 41% and 25% of {<Δ*E*_*j*_>} and {<Δ*q*_*j*_>} are related to {<Δ*IC*_*j*_>} (i.e., the square of the correlations shown in Table S4), respectively. Together with the correlation information in Fig. 2b, the analysis indicates that a stronger warming projection accompanies greater depletion of <Δ*IC*_*j*_>, and increased <Δ*E*_*j*_> and <Δ*q*_*j*_>.

The magnitude of a model’s <Δ*IC*_*j*_> relates to robust control climate characteristics. Figure 3 appraises the relationship between the zonal mean profiles of the 8 control climate variables and {<Δ*IC*_*j*_>} (outer panels). Warmer, rainier, more moist, and greater melting at the time of 4 × CO_2_ is associated with a control climate that is (a) much colder, particularly over the Antarctic, (b) much drier in the tropics but more moist in the northern extratropics, (c) less global cloudiness, (d) more ice/snow coverage, particularly in the Antarctic, (e) a stronger poleward energy and moisture transport, as indicated by positive values of the net radiative fluxes at the TOA in the tropics but negative values in the polar regions (Fig. 3e), and (f) less rainfall, particularly over the deep tropics. We term the control climate-WPS relationship described in (a–f) “type-A”. Subject to an anthropogenic radiative forcing, the “type-A” relationship predicts that a model with a colder (warmer) control climate state experiences larger (smaller) warming with a greater (lesser) melting of ice/snow, stronger (weaker) enhancement of rainfall and evaporation, and greater (smaller) increase in water vapor.

**Figure 4:**
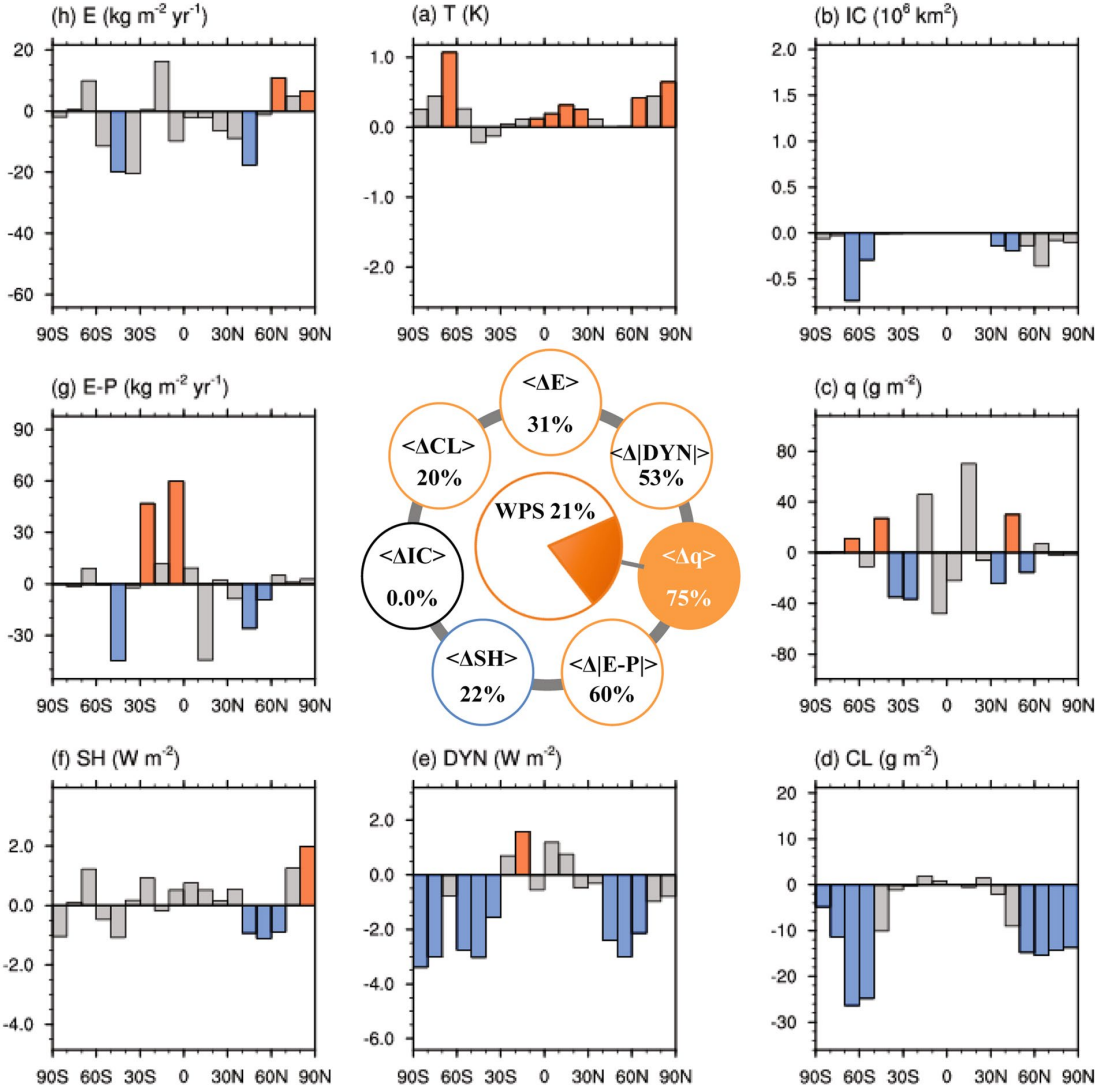
As in Fig. 3 except for the portion of each corresponding variable not correlated with the spread the total ice/snow area coverage response. All correlations are made with the remaining spread (75%) in the total column-integrated atmospheric water vapor response. The numbers inside the inner panel circle still represent the percentage of the spread, in the global mean changes of the eight key climate state variables that can be explained by the remaining portion of the spread in the total column-integrated atmospheric water vapor response.

The residual fields, obtained by removing the aforementioned relationships with {<Δ*IC*_*j*_>}, attribute the remaining WPS largely to the residual spread of {<Δ*q*_*j*_>}, denoted as {<Δ*q*_*j*_>^residual^} (Supplementary Fig. S5). Figure 4 (inner panel) shows that {<Δ*q*_*j*_>^residual^} accounts for 75%, 31%, and 21% of the total spreads of {<Δ*q*_*j*_>}, {<Δ*E*_*j*_>}, and {<Δ*T*_*j*_>}, indicating that the coupling between <Δ*q*_*j*_> and the other climate responses (Supplementary Table S4) remains discernable after removing the portion coupled with {<Δ*IC*_*j*_>} (Supplementary Fig. S5). The spreads of changes in poleward energy ({<Δ*|DYN*_*j*_ *|*>}) and latent heat ({<Δ*|E*_*j*_ − *P*_*j*_ *|*>}) transport possess particularly strong correlations with {<Δ*q*_*j*_>^residual^} (Fig. 4 and Supplementary Fig. S5). The residual spread signals that models with a greater increase in atmospheric water vapor, strengthened poleward energy transport as well as latent heat transport, and increased global cloud coverage warm more. Furthermore, there exists a robust relationship linking {<Δ*q*_*j*_>^residual^} and the remaining WPS to the residuals of the control climate spread (outer panels Fig. 4). In opposition to “type-A”, the residual control climate spread indicates that a warmer control climate with less ice coverage is associated with a greater increase in water vapor and larger warming. We term this control climate-WPS relation as “type-B”. The “type-A” relation accounts for the spread of {<Δ*IC*_*j*_>} and most of the WPS, while the “type-B” relation accounts for most of the remaining portion of the WPS and variance in {<Δ*q*_*j*_>}.

Considering control climate diversity, global mean surface temperature response, and climate feedbacks, a story emerges connecting WPS and control climate characteristics. The spreads of {<Δ*IC*_*j*_>} and {<Δ*q*_*j*_>} exhibit robust relationships with spreads in control climate characteristics, signaling inherited diversity. A “type-A” relationship indicates that a stronger (weaker) ice-albedo feedback corresponds to colder (warmer) control climate with more (less) ice coverage and greater (lesser) warming. Subsequently, a “type-B” relationship indicates that a stronger (weaker) water vapor feedback corresponds to a warmer (colder) control climate with less (more) ice/snow coverage and more (less) warming. For the type-A control climate, the spread in ice-albedo feedback strength drives the WPS, whereas the water vapor feedback spread drives the WPS for type-B. If type-A explained all of the WPS, we would expect a large inter-model spread for the ice-albedo feedback but a relatively small one for the water vapor feedback with the warming projection having a strong negative correlation to the control climate temperature. The converse would be true for the type-B scenario with the warming projection positively correlated to the control climate temperature. Therefore, these control climate-climate response relationships dictate a small chance of finding a model with an abnormally strong ice-albedo *and* water vapor feedback relative to other models. This control climate-climate feedback behavior also explains the weaker correlations between the WPS and the control climate diversity as compared to the climate feedback diversity. The opposing effects of control climate diversity on the ice-albedo and water vapor feedbacks obscures the relationship between WPS and control climate state diversity and has likely contributed to the lack of investigation into control climate-WPS relationships to understand uncertainty.

## Conclusions

Tracing the part of the WPS that is inherited from the diversity in the control climate state opens a new chapter to the WPS story, although it does not consider the scenario that different climate models can still have different global warming projections even if they have the same control climate state. Robust links between control climate, climate response, and the WPS provide supporting evidence for the emergent need to constraint model mean climate state for refining climate model projections^33, 34^. Specifically, WPS is related to control climate temperature and ice/snow cover in the Antarctic and the Southern Ocean supporting ongoing efforts to understand the underlying physical processes over this region^35, 36^. Unraveling relationships between the control climate states and climate responses show promise for reducing climate change uncertainty. Given the significant diversity among model control climates, this approach shows significant potential for narrowing the WPS. We do not challenge conventional thought on the importance of climate feedbacks, but enrich it by demonstrating that the inter-model spread in climate feedbacks partially inherits diversity from model control climates. New insights about the competing influences of the control climate on ice-albedo and water vapor feedbacks mark an important step forward. The control climate perspective allows us to probe deeper into the physics driving our climate models and their response. Hopefully, these new insights reopen an old and underexplored line of inquiry enabling us to pierce the unscathed armor surrounding WPS.

## Data and Methods

### Data

All data used in this study are derived from the monthly mean outputs of the CMIP5 1pctCO2 experiments. We only consider the first 140 years of simulated output fields. The information of model names and spatial resolutions of the 36 1pctCO2 experiments’ outputs is provided in Supplementary Table S1 and all data are archived and freely accessible at http://pcmdi9.llnl.gov/. We consider 31 of these models because (a) two of them were made without continuous increase of CO_2_ concentration after reaching the 2xCO_2_ and (b) three models did not provide the required outputs, such as 3D cloud fields.

### Key climate state variables and definitions of various averages

Eight key climate state variables are constructed at their native grids from the output fields listed in Supplementary Table S2. The definitions of the 8 key climate state variables and their units are provided in Supplementary Table S3. Because the native grids of different 1pctCO2 experiments have different spatial resolutions, we first calculate the zonal average of each key climate state variable at 18 10°-latitude wide bands, (*ϕ*_*0*_, *ϕ*_*0*_ + π/18) with *ϕ*_*0*_ = −π/2, −4π/9, $\cdots$, 4π/9, and π/2, according to

1 $${F}_{j}(n,{\varphi }_{0})=\frac{9}{{\pi }^{2}}{\int }_{{\varphi }_{0}}^{{\varphi }_{0}+\pi /18}\cos \,\varphi d\varphi {\int }_{0}^{2\pi }{f}_{j}(n,\varphi ,\lambda )d\lambda $$

where *λ* is longitude and *fj*(*n*) is one of the 8 key climate state variables (i.e., *n* = *1*, *2*, $\cdots$, *8*) at their native grids of the j^th^ 1pctCO2 experiment with *j* = *1*, *2*, $\cdots$, *31*.

We define the first 10-year average of ${F}_{j}(n,{\varphi }_{0})$ as the climate mean state of the j^th^ 1pctCO2 experiment, denoted as ${\overline{F}}_{j}(n,{\varphi }_{0})$. The ensemble mean of ${\overline{F}}_{j}(n,{\varphi }_{0})$ averaged over the 31 experiments is referred to as the ensemble mean climate state and the departure of ${\overline{F}}_{j}(n,{\varphi }_{0})$ for each *j* from the ensemble mean state measures the climate mean state diversity (or spread) of the j^th^ 1pctCO2 experiment, denoted as ${F}_{j}(n,{\varphi }_{0})$. The difference between the 10-year average of ${F}_{j}(n,{\varphi }_{0})$ taken from 130 to 140 years and ${\overline{F}}_{j}(n,{\varphi }_{0})$ corresponds to the (transient) climate response of ${F}_{j}(n,{\varphi }_{0})$ at the time of 4 × CO_2_, denoted as ${\rm{\Delta }}{\overline{F}}_{j}(n,{\varphi }_{0})$. The departure of ${\rm{\Delta }}{\overline{F}}_{j}(n,{\varphi }_{0})$ for each *j* from the ensemble mean of ${\rm{\Delta }}{\overline{F}}_{j}(n,{\varphi }_{0})$ averaged over the 31 experiments is denoted as ${\rm{\Delta }}{F}_{j}(n,{\varphi }_{0})$, measuring the uncertainty (or spread) in projecting the change/trend in the variable *F* by the j^th^ 1pctCO2 experiment. The global mean of ${\rm{\Delta }}{F}_{j}(n,{\varphi }_{0})$ is obtained by averaging ${\rm{\Delta }}{F}_{j}(n,{\varphi }_{0})$ over the 18 10°-latitude wide bands *ϕ*_0_, denoted as $< {\rm{\Delta }}{F}_{j}(n,{\varphi }_{0}) >$. We then we use “{X_j_}” to denote the series of 31 values of X_j_, where X_j_ can be ${F}_{j}(n,{\varphi }_{0})$ at *ϕ*_0_, or ${\rm{\Delta }}{F}_{j}(n,{\varphi }_{0})$ at *ϕ*_0_, or their global means.

### Analysis Procedures

All variance, correlation, and regression calculations are done for inter-model spreads (i.e., the corresponding calculations are done over *j*). The statistical significance of correlations is evaluated using the Student’s t-test. In the remaining discussion, we specifically use *n* = 8 for surface temperature *T* and the rest of n (*n* = *1*, *2*, *… 7*) for the other 7 variables. The following is the procedure for calculating the results shown in Figs 3 and 4.

- Identify $n\ne 8$ such that the correlation between $\{ < {\rm{\Delta }}{T}_{j} > \}=\{ < {\rm{\Delta }}{F}_{j}(n=8,{\varphi }_{0}) > \}$ and $\{ < {\rm{\Delta }}{F}_{j}({n}_{0},{\varphi }_{0}) > \}$ is maximum among all correlations of $\{ < {\rm{\Delta }}{T}_{j} > \}$ with $\{ < {\rm{\Delta }}{F}_{j}(n\ne 8,{\varphi }_{0}) > \}$.
- Calculate covariance of {*X*_*j*_} with $\{ < {\rm{\Delta }}{F}_{j}({n}_{0},{\varphi }_{0}) > \}$, denoted as ${\rm{cov}}(\{ < {\rm{\Delta }}{F}_{j}({n}_{0},{\varphi }_{0}) > \},\{{X}_{j}\})$, where *X*_*j*_ is one of the 152 variables (8 for $\{ < {\rm{\Delta }}{F}_{j}(n,{\varphi }_{0}) > \}$ and 8 × 18 for 8 $\{{F}_{j}(n,{\varphi }_{0})\}$ at the 18 latitude bands (*ϕ*_0_, *ϕ*_0_ + π/18). Then the correlation (“*a*”) and regression (“*r*”) coefficients are evaluated according to
  2 $$a(\{ < {\rm{\Delta }}{F}_{j}({n}_{0},{\varphi }_{0}) > \},\{{X}_{j}\})=\frac{{\rm{cov}}(\{ < {\rm{\Delta }}{F}_{j}({n}_{0},{\varphi }_{0}) > \},\{{X}_{j}\})}{\sqrt{{\rm{cov}}(\{ < {\rm{\Delta }}{F}_{j}({n}_{0},{\varphi }_{0}) > \},\{ < {\rm{\Delta }}{F}_{j}({n}_{0},{\varphi }_{0}) > \})\times {\rm{cov}}(\{{X}_{j}\},\{{X}_{j}\})}}$$
  3 $$r(\{ < {\rm{\Delta }}{F}_{j}({n}_{0},{\varphi }_{0}) > \},\{{X}_{j}\})=\frac{{\rm{cov}}(\{ < {\rm{\Delta }}{F}_{j}({n}_{0},{\varphi }_{0}) > \},\{{X}_{j}\})}{{\rm{cov}}(\{ < {\rm{\Delta }}{F}_{j}({n}_{0},{\varphi }_{0}) > \},\{ < {\rm{\Delta }}{F}_{j}({n}_{0},{\varphi }_{0}) > \})}$$
- Construct the residual spread of *X*_*j*_ according to,
  4 $${X}_{j}^{residual}={X}_{j}-r(\{ < {\rm{\Delta }}{F}_{j}({n}_{0},{\varphi }_{0}) > \},\{{X}_{j}\}) < {\rm{\Delta }}{F}_{j}({n}_{0},{\varphi }_{0}) > $$
  where $r(\{ < {\rm{\Delta }}{F}_{j}({n}_{0},{\varphi }_{0}) > \},\{{X}_{j}\}) < {\rm{\Delta }}{F}_{j}({n}_{0},{\varphi }_{0}) >$ is the part spread of *X*_*j*_ that can be explained by the spread of $\{ < {\rm{\Delta }}{F}_{j}({n}_{0},{\varphi }_{0}) > \}$.
- Replace $\{ < {\rm{\Delta }}{T}_{j} > \}$ with $\{ < {\rm{\Delta }}{T}_{j}{ > }^{residual}\}$ and {*X*_*j*_} with {${X}_{j}^{residual}$} and repeat the steps (a) – (c) until none of $\{ < {\rm{\Delta }}{F}_{j}(n,{\varphi }_{0}){ > }^{residual}\}$ for the remaining n statistically significantly correlated with $\{ < {\rm{\Delta }}{T}_{j}{ > }^{residual}\}$.

Note that $< {\rm{\Delta }}{F}_{j}({n}_{0},{\varphi }_{0}){ > }^{residual}\equiv 0$ since by definition, $r(\{ < {\rm{\Delta }}{F}_{j}({n}_{0},{\varphi }_{0}) > \},\{ < {\rm{\Delta }}{F}_{j}({n}_{0},{\varphi }_{0}) > \})\equiv 1$. It follows that we always end up with a distinct value of *n*_*0*_ in the new round of the steps (a,b).

Shown in Fig. S5 and the inner panels of Figs 3, 4 are the square of these correlation coefficients and outer panels of Figs 3–4 and S2–4 are $r(\{ < {\rm{\Delta }}{F}_{j}({n}_{0},{\varphi }_{0}) > \},\{{F}_{j}(n,{\varphi }_{0})\})\times \sqrt{{\rm{cov}}(\{ < {\rm{\Delta }}{F}_{j}({n}_{0},{\varphi }_{0}) > \},\{ < {\rm{\Delta }}{F}_{j}({n}_{0},{\varphi }_{0}) > \})}$ for n = 1, 2, … and 8.

### Online Content

Source Data, model variables, definitions and extended data display items are available in the online version of the paper, references unique to these sections appear only in the online paper.

## Electronic supplementary material

Supplementary Information
