# Supplementary material for: Inter-Model Warming Projection Spread: Inherited Traits from Control Climate Diversity
Source: Sci Rep. 2017 Jun 27;7:4300. doi: 10.1038/s41598-017-04623-7 (PMC5487336; doi:10.1038/s41598-017-04623-7)
Supplement: Supplementary file 1 — Supplementary Information [file 41598_2017_4623_MOESM1_ESM.docx]

**Supplementary Information for**

**Inter-Model Warming Projection Spread**: **Inherited Traits from Control Climate Diversity**

Xiaoming Hu^1^, Patrick C. Taylor^2^, Ming Cai^3,*^, Song Yang^1^, Yi Deng^4^, and Sergio Sejas^2^

^1^Department of Atmospheric Sciences, Sun Yat-sen University, Guangzhou, China

^2^NASA Langley Research Center, Climate Science Branch, Hampton, Virginia, USA

^3^Department of Earth, Ocean & Atmospheric Sciences, Florida State University, Tallahassee, Florida, USA

^4^School of Earth and Atmospheric Sciences, Georgia Institute of Technology, Atlanta, Georgia, USA

^*^ To whom correspondence should be addressed**.** Department of Earth, Ocean and Atmospheric Science, Florida State University, 1017 Academic Way, Tallahassee, FL, 32306. E-mail: [mcai@fsu.edu](mailto:mcai@fsu.edu)

Table S1: CMIP5 models and simulations used in this study.

| Simulation  No. | | CMIP5  Model Name | 1pctCO_2_ Simulation |
| --- | --- | --- | --- |
|  | 1 | bcc-csm1-1-m | r1i1p1 |
|  | 2 | bcc-csm1-1 | r1i1p1 |
|  | 3 | CanESM2 | r1i1p1 |
|  | 4 | CCSM4 | r1i1p1 |
|  | 5 | CESM1-BGC | r1i1p1 |
|  | 6 | CESM1-BGC | r1i1p2 |
|  | 7 | CESM1-CAM5 | r1i1p1 |
|  | 8 | CMCC-CM | r1i1p1 |
|  | 9 | CNRM-CM5-2 | r1i1p1 |
|  | 10 | CNRM-CM5-2 | r1i1p2 |
|  | 11 | CNRM-CM5-2 | r1i1p3 |
|  | 12 | CNRM-CM5 | r1i1p1 |
|  | 13 | CSIRO-Mk3L-6-0 | r1i1p1 |
|  | 14 | GFDL-CM3 | r1i1p1 |
|  | 15* | GFDL-ESM2G | r1i1p1 |
|  | 16 | GFDL-ESM2G | r1i1p2 |
|  | 17* | GFDL-ESM2M | r1i1p1 |
|  | 18 | GFDL-ESM2M | r1i1p2 |
|  | 19 | GISS-E2-H | r1i1p1 |
|  | 20 | GISS-E2-H | r1i1p2 |
|  | 21 | GISS-E2-H | r1i1p3 |
|  | 22 | GISS-E2-R | r1i1p1 |
|  | 23 | GISS-E2-R | r1i1p2 |
|  | 24 | GISS-E2-R | r1i1p3 |
|  | 25 | inmcm4 | r1i1p1 |
|  | 26* | IPSL-CM5A-LR | r1i1p1 |
|  | 27* | IPSL-CM5A-MR | r1i1p1 |
|  | 28* | IPSL-CM5B-LR | r1i1p1 |
|  | 29 | MIROC-ESM | r1i1p1 |
|  | 30 | MIROC5 | r1i1p1 |
|  | 31 | MPI-ESM-LR | r1i1p1 |
|  | 32 | MPI-ESM-MR | r1i1p1 |
|  | 33 | MPI-ESM-P | r1i1p1 |
|  | 34 | MRI-CGCM3 | r1i1p1 |
|  | 35 | NorESM1-M | r1i1p1 |
|  | 36 | NorESM1-ME | r1i1p1 |

Table S2. Variables considered in this study.

| Variable Name | Abbreviation | Units |
| --- | --- | --- |
| Condensed Water Path | clwvi | kg m^-2^ |
| Evaporation | evspsbl | Kg m^-2^s^-1^ |
| Precipitation | pr | kg m^-2^s^-1^ |
| Specific Humidity | hus | kg kg^-1^ |
| Surface Downwelling Longwave Radiation | rlds | W m^-2^ |
| Surface Downwelling Shortwave Radiation | rsds | W m^-2^ |
| Surface Temperature | ts | K |
| Surface Upward Latent Heat Flux | hfls | W m^-2^ |
| Surface Upward Sensible Heat Flux | hfss | W m^-2^ |
| Surface Upwelling Longwave Radiation | rlus | W m^-2^ |
| Surface Upwelling Shortwave Radiation | rsus | W m^-2^ |
| Toa Incident Shortwave Radiation | rsdt | W m^-2^ |
| Toa Outgoing Shortwave Radiation | rsut | W m^-2^ |
| Toa Outgoing Longwave Radiation | rlut | W m^-2^ |

Table S3. The 8 key climate state factors, their definitions, and units

| Variable | Abbreviation | Definition* | Units |
| --- | --- | --- | --- |
| Surface Temperature | **T** | **$T=(ts)-273.15$** |  |
| Vertically Integrated Water Vapor Content | **q** | **$q=\int_{100hPa}^{1000hPa} (hus)\times100\frac{dp}{g}$** | kg m^-2^ |
| Vertically Integrated Cloud Water/Ice Content | **CL** | **$q=\int_{100hPa}^{1000hPa} (clwvi)\times100\frac{dp}{g}$** | kg m^-2^ |
| Area Covered By Ice/Snow* | **IC** |  | $10^{6}m^{2}$ |
| Net Downward Radiative Fluxes at the Top of the Atmosphere** | **DYN** | $\begin{matrix} TOA=rsdt-rsut-rlut \\ SRF=rsds-rsus+rlds-rlus-hfss-hfls \\ DYN=TOA-SRF \end{matrix}$ | W m^-2^ |
| Evaporation | **E** | $E=(evspsbl)$ | kg m^-2^ s^-1^ |
| Evaporation Minus Precipitation*** | **E − P** | $E-P=(evspsbl-pr)$ | kg m^-2^ s^-1^ |
| Surface Sensible Heat Flux | **SH** | $SH=(hfss)$ | W m^-2^ |

* Surface albedo is defined as the ratio of the annual mean upward (reflected) solar radiative fluxes at the surface to the annual mean downward solar radiative fluxes at the surface.

** Regions of positive values of *DYN* correspond to divergence of the vertically integrated energy transport by atmospheric circulations whereas negative values correspond to convergence of the vertically integrated energy transport by atmospheric circulations.

*** Similar to “DYN”, positive values of *L(E – P)*, where *L* is latent heat constant, represent divergence of the vertically integrated latent heat transport by atmospheric circulations whereas negative values correspond to convergence of the vertically integrated latent heat transport by atmospheric circulations.

Table S4. Cross-correlation coefficients among global mean changes of the 8 key climate variables (bold indicates a 95% confidence level of t-test)

| corr | {<ΔT_j_>} | {<Δic_j_>} | {<Δq_j_>} | {<ΔE_j_>} | {<Δ\|dyn\|_j_>} | | {<Δ\|e-p\|_j_>} | {<ΔSH_j_>} | {<ΔCL_j_>} |
| --- | --- | --- | --- | --- | --- | --- | --- | --- | --- |
| {<ΔT_j_>} | **1** | **−0.83** | **0.82** | **0.85** | 0.15 | 0.28 | | **−**0.29 | 0.1 |
| {<Δic_j_>} |  | **1** | **−0.5** | **−0.64** | 0.15 | **−**0.01 | | 0.02 | 0.03 |
| {<Δq_j_>} |  |  | **1** | **0.80** | **0.56** | **0.67** | | **−0.41** | **0.37** |
| {<ΔE_j_>} |  |  |  | **1** | 0.18 | **0.45** | | **−0.37** | 0.16 |
| {<Δ\|dyn\|_j_>} |  |  |  |  | **1** | **0.63** | | **−**0.31 | 0.18 |
| {<Δ\|e−p\|_j_>} |  |  |  |  |  | **1** | | **−**0.19 | **0.47** |
| {<Δsh_j_>} |  |  |  |  |  |  | | **1** | **−0.47** |
| {<ΔCL_j_>} |  |  |  |  |  |  | |  | **1** |


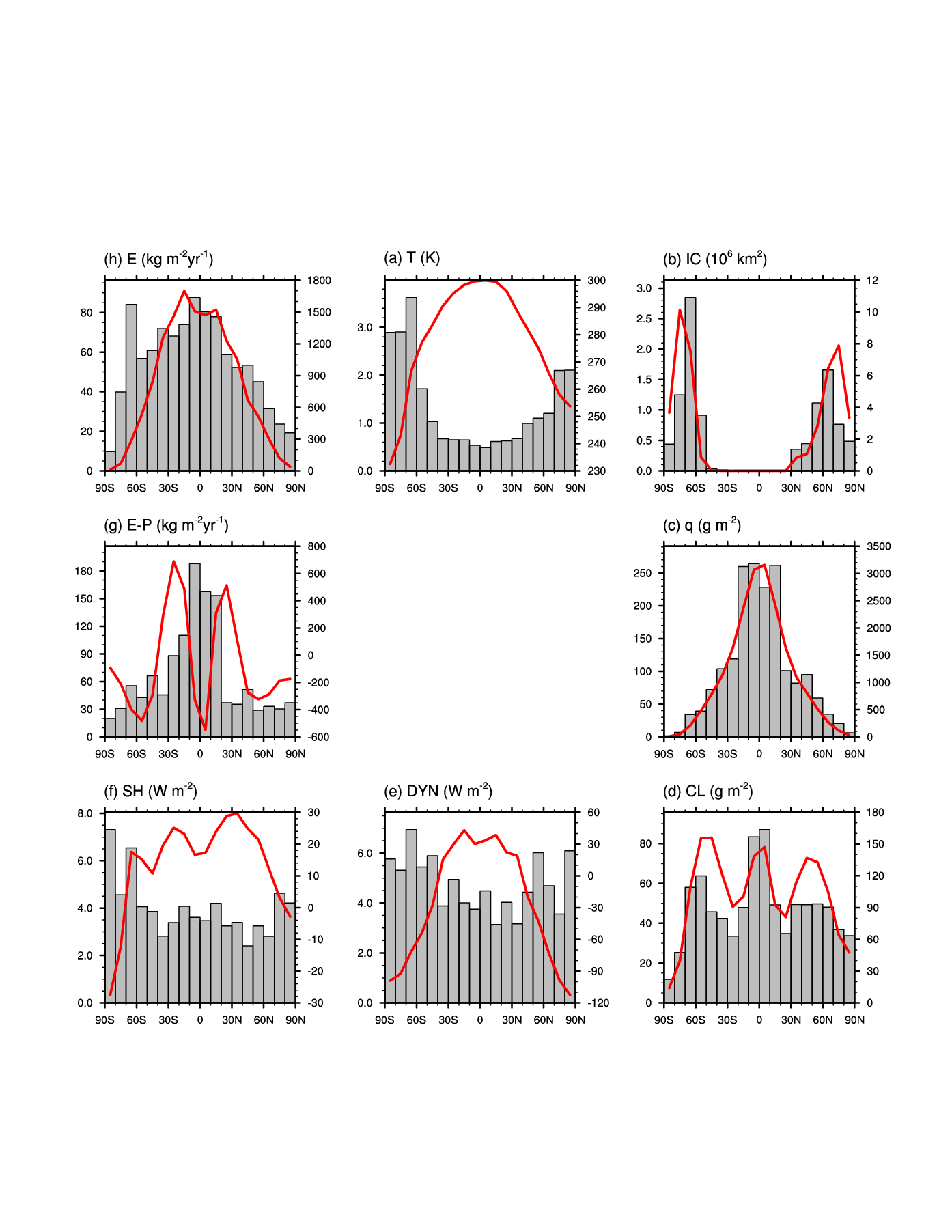


Figure S1. Latitudinal profiles of the 31-member ensemble means (red lines and the axes on the right) and their spreads defined as the standard deviation with respect to their corresponding ensemble means (bars and the axes on the left) of the zonal means of the 8 key state climate variables in the control climate state. (a) surface temperature (*T* in units of K), (b) total area covered by ice/snow (*IC* in units of km^2^), (c) vertically integrated atmospheric water vapor content (*q* in units of g m^-2^), (d) vertically integrated cloud water/ice content (*CL* in units of g m^-2^), (e) net downward radiative fluxes at TOA which measures the strength of the total atmosphere-ocean energy transport (*DYN* in units of W m^-2^), (f) surface sensible heat flux (*SH* in units of W m^-2^), (g) difference between surface evaporation rate and precipitation rate (*E − P* in units of kg m^-2^ yr^-1^), and (h) evaporation rate (*E* in units of kg m^-2^ yr^-1^).


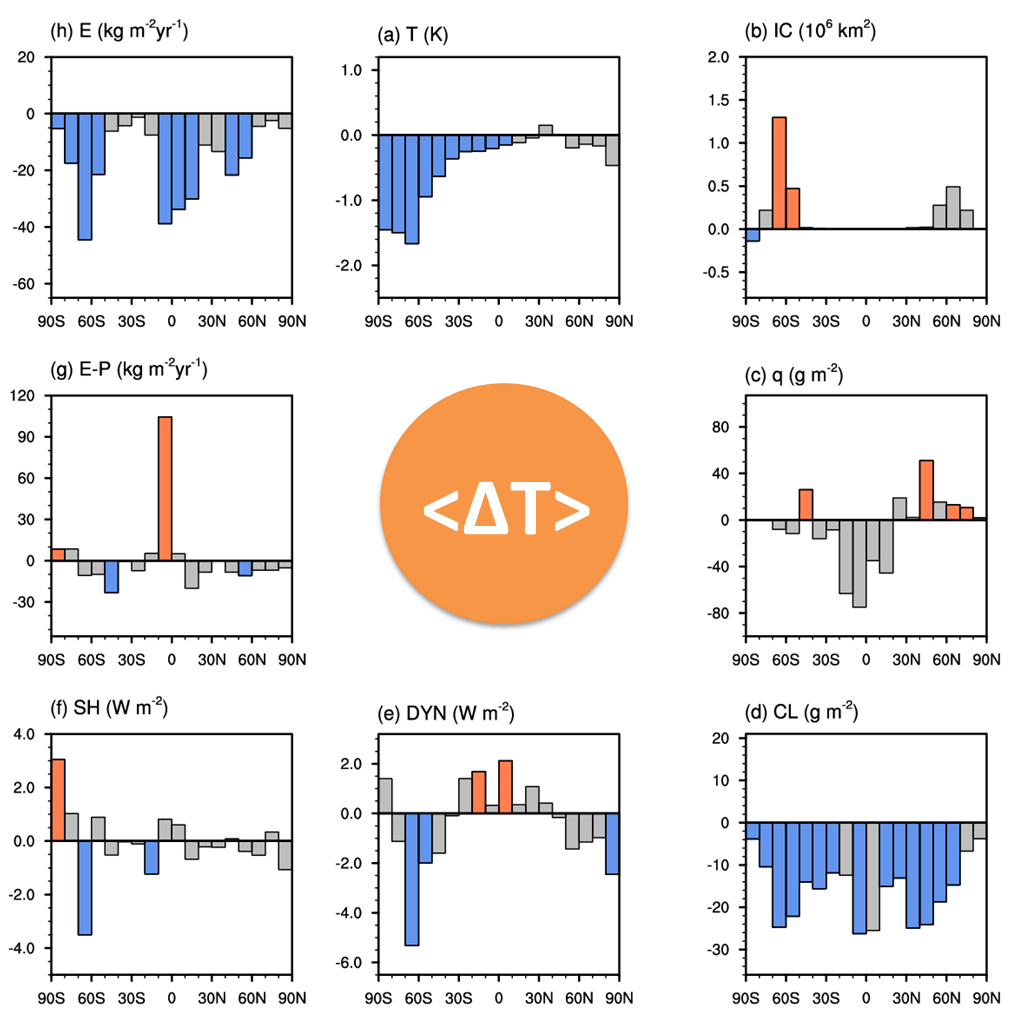


Figure S2. Latitudinal profiles of the regressed spreads of the zonal mean control climate states (a-h) against the spread of the global mean surface temperature change. (a) surface temperature (T in units of K), (b) total area covered by ice/snow (IC in units of km^2^), (c) vertically integrated atmospheric water vapor content (q in units of g m^-2^), (d) vertically integrated cloud water/ice content (CL in units of g m^-2^), (e) net downward radiative fluxes at TOA which measures the strength of the total atmosphere-ocean energy transport (DYN in units of W m^-2^), (f) surface sensible heat flux (SH in units of W m^-2^), (g) difference between surface evaporation rate and precipitation rate (E-P in units of kg m^-2^ yr^-1^), and (h) evaporation rate (E in units of kg m^-2^ yr^-1^). Orange and blue colored (grey) bars indicate the correlation coefficients (do not) exceed 90% confidence level.


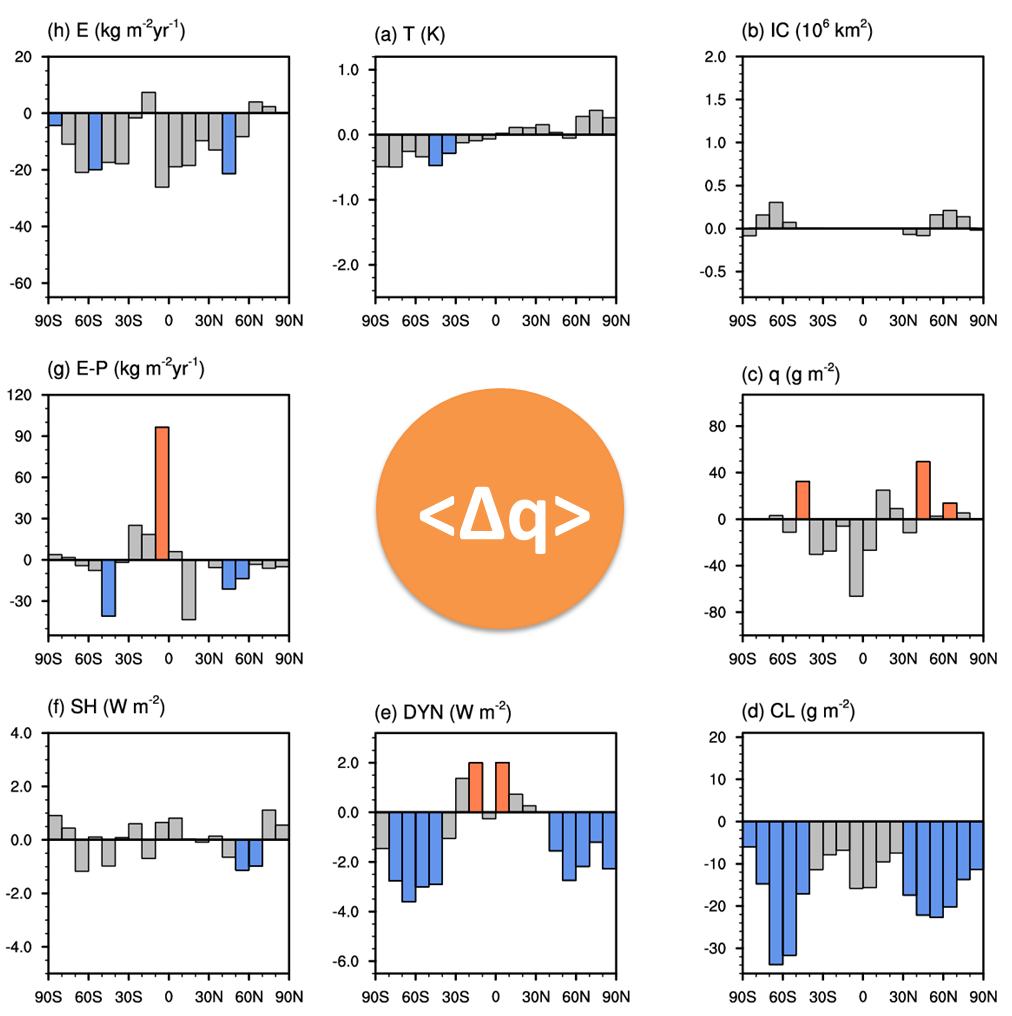


Figure S3. Same as Figure S2 except the regressions are made against the spread of the global mean atmospheric water vapor change.


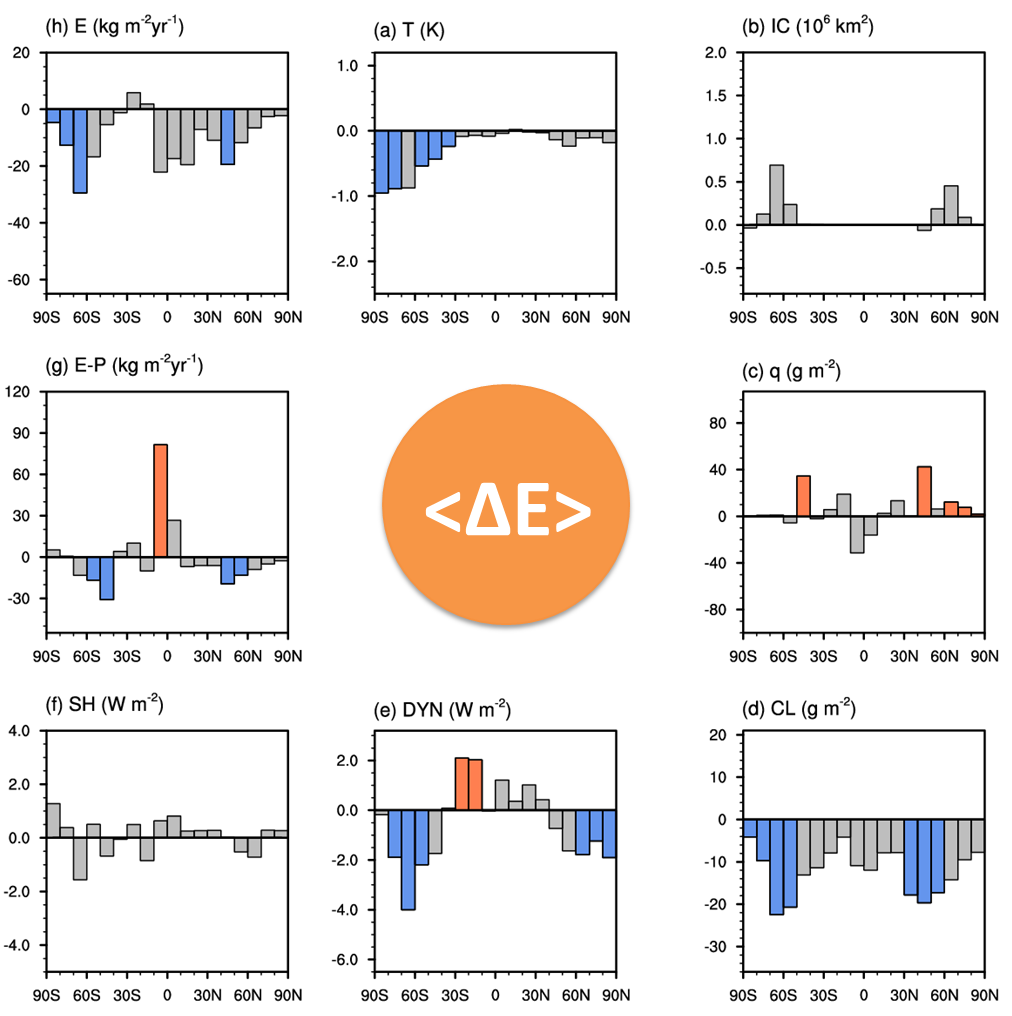


Figure S4. Same as Figure S2 except the regressions are made against the spread of the global mean evaporation change.


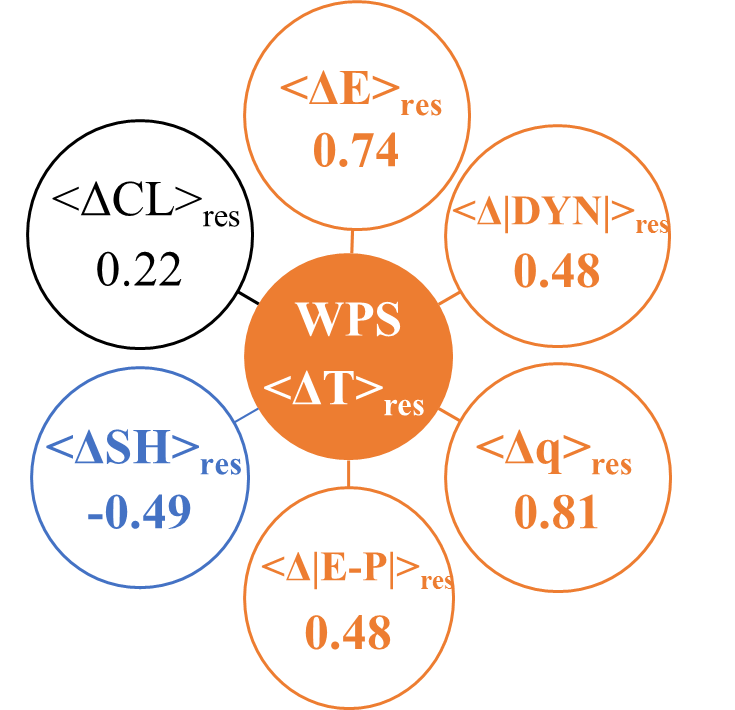


Figure S5. Correlation coefficients between the residual in the warming projection spread and the residual spreads in the global mean changes of other 7 key climate variables. A residual spread is defined as the portion of the spread that cannot be explained by the spread in the change of the total area covered by ice/snow. Orange and blue colored (black) numbers/circles indicate the correlation coefficients (do not) exceed 90% confidence level.
